# Determinants of breast cancer early detection for cues to expanded control and care: the lived experiences among women from Western Kenya

**DOI:** 10.1186/s12905-018-0571-7

**Published:** 2018-06-01

**Authors:** Joyce Kisiangani, Joyce Baliddawa, Pamela Marinda, Hillary Mabeya, Joseph K. Choge, Eric Onyango Adino, Christopher Khayeka-Wandabwa

**Affiliations:** 1 The Aquaya Institute, Nairobi, 00505 Kenya; 2 School of Public Health, Department of Epidemiology and disease control, Moi University, P.O. Box 3900, Eldoret, Kenya; 3 Department of Food Science and Nutrition, School of Agricultural Sciences, The University of Zambia, Lusaka, Zambia; 4 Gynocare Fistula Centre, Eldoret Hospital Lane, P.O. BOX 2326-30100, Eldoret, Kenya; 5 University of Kabianga, P.O. Box 2030-20200, Kericho, Kenya; 6 East African Breweries Limited, Nairobi, Kenya; 7 School of Pharmaceutical Science and Technology (SPST), Health Science Platform, Tianjin University, 92 Weijin road, Nankai District, Tianjin, 300072 People’s Republic of China; 8 African Population and Health Research Center (APHRC), P .O. Box 10787-00100, Nairobi, Kenya

**Keywords:** Breast cancer, Early breast cancer screening, Focused group discussions (FGD), Key informant interviews

## Abstract

**Background:**

Estimately, 70–80% of cancer cases are diagnosed in late stages in Kenya with breast cancer being a common cause of mortality among women where late diagnosis is the major ubiquitous concern. Numerous studies have focused on epidemiological and health policy dynamics essentially underestimating the determining factors that shape people’s choices and cues to health care service uptake. The study sought to evaluate the knowledge, attitude and health seeking behavior towards breast cancer and its screening in a quest to explain why women present for prognosis and treatment when symptomatic pointers are in advanced stages, impeding primary prevention strategies.

**Methods:**

Eight focus groups (6–10 members per group) and four key informant interviews were conducted among adult participants from rural and urban settings. Sessions were audio-recorded and transcribed. A thematic analysis of the data was based on the concepts of the health belief model. Data analysis was conducted using NVIVO10.

**Results:**

Most women perceived breast cancer as a fatal disease and conveyed fear of having early screening. Rural women preferred self-prescribed medications and the use of alternative medicine for long periods before presenting for professional care on suspicion that the lump is cancerous. Accessibility to equipped health facilities, lack of information to establish effective follow-up treatment and low-income status were underscored as their major health seeking behavior barriers whereas, urban women identified marital status as their main barrier. Key informant interviews revealed that health communication programs emphasized more on communicable diseases. This could in part explain why there is a high rate of misconception and suspicion about breast cancer among rural and urban women in the study setting.

**Conclusions:**

Creating breast cancer awareness alongside clear guidelines on accessing screening and treatment infrastructure is critical. It was evident, a diagnosis of breast cancer or lump brings unexpected confrontation with mortality; fear, pain, cultural barriers, emotional and financial distress. Without clear referral channels to enable those with suspicious lumps or early stage disease to get prompt diagnosis and treatment, then well-meaning awareness will not necessarily contribute to reducing morbidity and mortality.

**Electronic supplementary material:**

The online version of this article (10.1186/s12905-018-0571-7) contains supplementary material, which is available to authorized users.

## Background

Cancer is the third highest cause of mortality in Kenya after infectious and cardiovascular diseases. Leading cancers are breast and cervical for women [1, 2]. Seventy to 80 % of cancer cases are diagnosed in late stages. Like many other Non-Communicable Diseases (NCDs) breast cancer progresses slowly, degenerates to devastating disabilities and the management costs are high if not timely diagnosed and treated. There is better prognosis, greater chances of successful treatment and high survival rates when detected at early stages. Methods such as clinical breast exams (CBE), mammograms and breast self-examinations (BSE) have been used as main approaches [3, 4].

Health care access is considered a multidimensional concept encompassing both financial and non-financial dimensions [5, 6]. It has broadly been defined as the degree of fit between a patient’s socioeconomic characteristics, the health system, and health services organization [5, 6]. The five core components of access that have been outlined are: acceptability, affordability, accessibility, accommodation, and availability [5–9]. In Kenya, tremendous gains have been made in the recent past on affordability, accommodation, and availability of cancer screening and treatment services whereas much more effort still needs to be put on accessibility and acceptability [8, 9]. Compelling findings on breast cancer in Kenya still show that most women are not aware of signs and symptoms of breast cancer due to cultural diversity views and limited education and awareness programs with a lag on clear referral channels information empowerment [8, 10–14]. This considers the fact that, there are varied underlying information and awareness factors among Kenyan women on early cancer screening and on why and under what conditions they would take action towards medical attention for prevention or early screening and treatment [4, 15, 16].

As studies shed more understandings on the the risks and benefits of early breast cancer screening, indepth understanding of women perceived risk and barriers have become integral [13, 17, 18]. The insights can help influence women choice of approaching early screening and treatment options or risk-reduction strategies and effective follow-up treatment [17–19] in a targeted approach that resonate with their gendered socio-cultural role; their perceived susceptibility, severity, benefits and perceived health needs [6, 20]. The study thus generates and enhances the pool of evidence that would aid inform the development of local cancer information, education and communications (IEC) tailored for communities in Kenya incorporating approaches that fully engage the target populations [21]. As a result, potentially promote optimization of the existing and upcoming national health systems for cancer management under the vision 2030 and beyond as envisaged under the cancer awareness, community engagement plan [6, 14, 21–23].

## Methods

### Study design

**Fig. 1:**
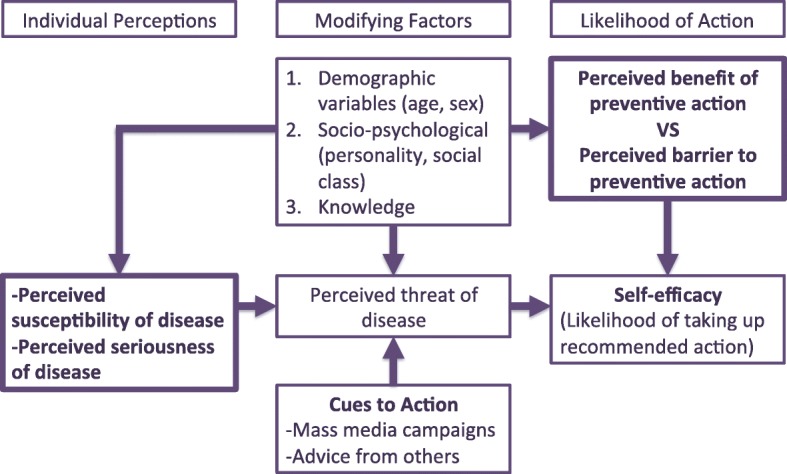
Conceptual framework for the qualitative interview. Adapted from [30, 31]

The qualitative research was conducted between November 2013 and March 2014. A qualitative design using focus group discussion (FGD) and key informant interview (KII) methods was applied as an ideal approach to explore perceived motivators and barriers to healthy behaviours [24]. The items in the FGD and KII were developed based on the concepts of the health belief model [16, 25–29]. Interview guide for the focus group discussions and in-depth interviews (key informants) are as provided (see Additional file 1). The concepts included; percieved severity of breast cancer, perception of susceptibility to breast cancer, perceived benefits of breast cancer early detection measures, perceived barriers to breast cancer early detection measures, self-efficacy and cues to action. The conceptual framework for the qualitative interview was adapted as earlier reported [30, 31] and is shown in Fig. 1. This approach was selected because FGDs and KIIs can be undertaken in naturalistic settings which may stimulate more openness and candor [32, 33]. Also the group interaction has the capability to elicit information and insights that are less accessible during individual interviews [34]. Probing by the moderator allowed in depth exploration of unanticipated issues as well as an opportunity to clarify and enhance understanding of responses [33].

### Study setting and participants

Kakamega is a County in Western Kenya lying about 30 KM north of the Equator. The county has a population of about 1.7 million (KNBS, 2010) with a population density of about 544 people per square kilometer and is Kenya’s second most populous county after Nairobi [35]. According to the 2009 census, 15.2% of the population lives in urban areas. Of the approximately 1.7 million people,48% are male and 52% female. The population is relatively poor with a poverty rate of 53% [35]. The main health facilities in the county and its environs are: Kakamega General Hospital, Central Maternity and Nursing Home, Butere district hospital, Malava district hospital, Mumias district hospital, Matungu, Manyala and Navakholo sub-district hospitals, Kima Mission Hospital and Kimilili District Hospital.

The researcher divided the 12 constituencies into two groups; urban and rural using Kakamega First County Development Plan, 2013. Map of the study areas, Kakamega couty constituencies and study locations (see Additional file 2). Urban groups include: Mumias West, Lurambi, Shinyalu, Mumias East and Butere whereas Rural groups comprise: Matungu, Khwisero, Malava, Ikolomani, Navakholo, Lugari and Likuyani. Two constitntuencies for rural settings (Ikolomani and Likuyani) and urban settings(Mumias West and Lurambi) respectively were randomly selected. Eligible participants were selected using a purposive sampling method. Thus, for the FGDs, homogenity on particular characteristics was considered; (in this case it was for gender, age and rural or urban) and recruited from the communities of the randomly selected constituencies. Convenience sampling (number of locations and sub locations per consitutency for more objective representation) was utilized for selection of the FGDs members by the researchers with the help of local leaders (chiefs and sub-chiefs) through their documented community governance records. Thus, the FGDs were conducted in two groups: young women of age group 18–35 and older women of age group 36–60 emanating from either urban or rural concsitituency. Hence, 72 participants all above 18 years were interviewed, and comprised of four key informants and 68 members of 8 FGDs with between 6 and 10 participants per FGD. Two (2) focus groups were drawn from each constituency. For each set of 2 groups, 1 FGD constituted women of age 18–35, and another of women of age 36–60. One (1) key informant was drawn from each constituency. Key Informants were the four District Public Health Nurses (DPHN) that were from the four constituencies where the FGDs were done. The DPHN were considered because, they interact with a significant proportion of women in these communities and therefore considered to be more knowledgeable of what the women face in obtaining early breast cancer screening. Consented participants were allocated to a focus group session or Key informant interviews (KII) based on the respective eligibility cirteria.

### Data collection

The items in the focus group discussion interviews and key informant interviews were developed based on the concepts of the health belief model (as above-mentioned and detailed). The concepts were pretested with small groups outside the target study area and revisions made where necessary. Information from KII (the first one to be conducted) helped inform some of the questions to ask in FGD. Thus, gaining further understanding on the validity of DPHN perspective in resonance to the population they attend to and considering they often provide inputs in appraising government health sector operational guidelines and policies which have a direct bearing on the populations they serve. Focus groups lasted between 43 to 65 min. Rural focus groups were conducted in Swahili while discussions with urban women took place in Swahili and English. The items in FGD interviews were initially formulated in English then translated into Swahili for the use in the different study locations. Data collected in Swahili versions were translated back to English to ensure consistitency with the data collected in English versions. The five-phase cycle of compiling, disassembling, re-assembling (and arraying), interpreting and concluding were used to process the data as earlier described [36].

### Data analysis

All the data from the KIIs and FGDs were then uploaded to the Computer Assisted Qualitative Data Analysis Software (CAQDAS) QSR Nvivo10 for windows for management and analysis. Coding was done as earlier described [36] with level 1 to level 3 coding with the following major emerging categories from the level three coding: health seeking behavior of breast cancer and its screening, knowledge of breast cancer and its screening, attitude towards breast cancer and its screening and breast health promotional strategies. Comparisons between the four groups; urban rural, 18–35 year old women and those aged 36–60 years were made as categories emerged. Findings were reported per the themes/topics that emerged.

## Results

Findings from the focus group discussions and key informant interviews are presented per the main categories from level 3 coding that emerged.

### Category one: Health seeking behavior of breast cancer and its screening

Sixty-eight women aged 18–60 years (age: 18–35: 36 and 36–60: 32) from the selected rural and urban constituencies of Kakamega county Kenya participated in eight focus groups (average 8.5 participants per group). Of these 63 (92.6%) were Christians and 5 (7.4%) were Muslims. Participants were predominantly married (72.1%). The educational level of the respondents varied considerably; 45.6% had primary education, 35.3% secondary, 11.8% middle level college training and 2.9% university degree while the rest had no formal schooling. There were 32 women from the rural setting and 36 urban. The average family size was 6 persons, with an average of 4 children per woman. All working women had government medical cover, the National Health Insurance Fund (NHIF) through their jobs, granting coverage for health service use at governmental facilities for themselves and their dependents. Despite having NHIF cover, the women expressed a generalized preference for private health care providers. They however explained that, their choice to use public rather than private services was often mediated by a series of social, economic, and geographical considerations. None of the women had private health insurance cover instead self-help savings and credit groups, commonly known as *chamas* were common source of building financial capacity and borrowing among the women. The vast majority of women rated their health status as good.

There were disparities between the urban and rural when it came to health seeking behavior barriers (see Additional file 3). Most urban women (from all the 4 urban FGDs) identified marital status as their major barrier to early breast cancer screening. Specifically, young-urban women explained that married women have to consult and at times get not only advice but also permission from their husbands before seeking any medical help or undertaking social commitments that has an impact on their health. An urban woman from group 7 to symbolically contextualize the challenge depth narrated:

*“Some men do not allow their women to go to the hospital. Most men do not even allow their women to go out of the homestead and ‘fetch water for domestic use’, how then will they even allow women to go for cancer screening at the hospital?”*

They went further to explain that as the head of the home, some husbands would have difficulties in allowing their women to go for cancer screening as it is a disease that is associated with terminal health complications equating it to be even more worse than HIV/AIDS and getting to know makes it even more hard to live with the new reality. Contrary, rural women (up to 75% of rural participants) identified long distance to health facility, lack of information on breast cancer and its screening and low-income status as major health seeking behavior barriers. An older woman from rural group 4 said:

*“Ignorance and lack of information on the importance of cancer screening hinders women from seeking early breast cancer screening”.*

A younger woman from rural group 1, said:

*“The long distance to the cancer facility would cost a lot of transport fare that I cannot afford and this makes it difficult for me.”*

An interview with Key informant revealed that the health centres and hospitals do not have enough nurses to perform regular breast cancer screening. She narrated:

*“The number of staff in the hospitals should be increased. Sometimes you will find there is only one nurse who has to do everything in a hospital. When a woman comes in for a BCS, the nurse is most likely to attend to the patients whose lives are at risk first. There should be devolution. The number of staff should be increased.”*

Through indepth discussions it was evident cultural religion orientation was a contributing barrier among rural women compared to their urban counterparts. A rural participant from rural group 3 said;

*“Some women go to churches that believe in prayers for healing, the churches do not allow their followers to go to the hospital. Such women do not go for breast cancer screening.”*

The study further revealed that most women are skeptical of having early breast cancer screening as attributed to fear of getting a positive breast cancer diagnosis; stigmatization associated with it and breast cancer related cultural beliefs alongside misconceptions as well as the, what next? doubts. The action taken when a woman realized she had a breast lump was influenced by the community perception of the origin of the disease for the older urban participants, conventional practices and beliefs for most rural participants and level of knowledge by younger-rural women. Most urban women described the community perception and association of breast cancer to promiscuity, infidelity, and equating it to HIV/AIDS thus hindering women who suspected a lump in their breast was cancerous from seeking early treatment in hospitals. An older woman from the urban group 8 narrated:

*“If a woman is known to be sleeping around with many men, and she discovers that she has breast cancer, she will prefer not to go and seek treatment because she is afraid that she might go to the hospital, get tested for HIV and told that she has AIDS.”*

Rural women on the other hand preferred using herbal remedies, self-medicating with painkillers or going to traditional medicine men for complementary and alternative remedy when they suspected they had a cancerous lump in their breast. A woman from rural group 3 said:

*“Women believe it is a sore, so we take traditional herbs called “miyeka”.”*

We also established that younger-rural participants and older participants were not aware or clear of simple methods such as breast self-examination or where they could get early breast-screening services. Several older participants asked to be taught how to detect a lump in their breast while younger women explained that they were not confident on performing breast self-examination on themselves. A young participant explained in frustration how nurses instructed them to palpate their breast but she did not understand how palpation was done or what the nurses meant by palpate. When asked how to check for lumps in their breast, a participant from urban group 8 said:

*“We do not know. You should teach us on how one knows that they have breast cancer.”* This is despite the fact that health centres in Kakamega County schedule a day in a month for breast health education. A key informant from a rural group explained that most facilities in the county conduct breast screening at facility level once a month. However, she also added that it was likely that women did not know that they could access such services for free due to lack of information.

### Category two: Knowledge of breast cancer and its screening

Younger participants seemed to have a better comprehension of breast cancer, its early manifestations, early breast cancer screening and predispositions’ to the disease compared to older participants. A participant from rural group 1 defined breast cancer as:

*“I know they are cells that multiply in the breast leading to death.”*

Specifically, young urban participants seemed more knowledgeable about lifestyle issues that predispose individuals to breast cancer. They mentioned smoking, too much sugar and salt in food, use of bleaching pills and self-medicated pills. A woman in urban group 7 said:

*“I know that it is brought about by smoking and using a lot of fat in your food.”*

Other misconceptions across the women included being born with “risky bugs” in their breasts, breast cancer being a consequence of having HIV and prostitution as well as surgery of the breast being associated with breast cancer status and death.

### Category three: Attitudes of women toward breast cancer and its screening

Most women were concerned of breast cancer and were overwhelmingly convinced that it is a serious terminal disease with no cure. Terms such as ‘deadly and death’ were commonly used in the description of breast cancer. A participant in rural group 2 said:

*“Breast cancer is a death sentence.”*

Another participant in rural group 1 said:

*“I understand that it has no cure. It is an incurable disease.”*

Several participants also described the perceived seriousness of the disease as extremely dangerous and incurable. A young woman in urban group 5 stated:

*“The women in the community believe that breast cancer is a disease that does not have a cure and so will not bother to go to the hospital.”*

Participants were also afraid of CBE. A participant from urban group 8 narrated:

*“I do not think going for breast cancer screening is a necessity. One woman that I know went for breast cancer screening and was told that she had it and that her breast had to be removed... When her breast was cut, the cancer spread throughout her body and following that, I and many of my friends who knew her can’t go to the hospital.”*

Some urban participants seemed well aware of the benefits of early breast cancer screening. They noted that knowing their breast cancer status early would result to the early treatment of the disease and this would increase the chances of survival. They also explained how early screening uptake reduces the high cost of treatment of the disease if it is detected early. A woman from urban group 6 said:

*“If I am found with cancer at an early stage, it means that I will not spend much on the treatment of the disease. It will be cheaper for me.”*

Despite this knowledge among some of the participants, they were still sceptical of having early breast cancer screening as it would lead to psychological stress, depression and even early death unlike when they were not aware.

### Category four: Breast health promotion strategies

Most of the participants had very limited knowledge of breast health awareness programs. Participants could only mention programs aimed at communicable diseases (Malaria and HIV) awareness creation. Participants from rural group 2 said:

*“There is no much emphasis on breast cancer awareness campaigns.”*

The participants pointed out the reason why they do not have much information on breast cancer was because it was not being given as much prominence as other diseases like malaria per their views on assessment of public health information availed to them most of the times. An interview with a key informant from urban setting revealed that if participants were given information on breast cancer and its screening; there would be rapid uptake of early breast cancer screening among women. She said:

*“In this community, if a woman does not know the importance of early breast cancer screening, they will not go for screening. But after they have been taught on the importance, many of them flock to the screening rooms and get screened for cancer. An example is cervical cancer. Most women have had a pap smear done on them because there have been consistent seminars and campaigns on the importance of doing a pap.”*

An urban participant in group 5 when asked what measures can be put in to motivate women to go for early screening said:

*“If they gave us information on breast cancer, it would motivate me to go for screening.”*

The participants also asked for free and regular breast cancer screening services to be made available not only in mother-child health clinics but also in the overall hospitals services. When the women were asked on the best channel of communication of information on breast cancer and its screening, urban participants suggested the use of media, and mother-child health clinics. Most young participants suggested use of community health workers (CHW), social media and text messages as the best avenues for the communication of information. Rural participants suggested the use of community health workers. A rural participant said:

*“Through seminars- they should take CHW’s on seminars, and when they come they should educate the community on breast cancer by doing door to door education.”*

Older women suggested use of door to door education, churches, village meetings (*barazas*) and village chief’s as the best channel for communication of information on breast cancer and its screening. Key informant 2 suggested:

*“Verbal health education- use of wall charts, the mass media, chief barazas and use community leaders (church leaders and chiefs to talk to them).”*

## Discussion

In the presented findings, there were disparities between urban and rural women when it came to knowledge, attitude and health seeking behavior towards breast cancer and its screening. Whereas urban women identified concerns such as partner related consensus, rural women mostly identified the lack of information, long distance to health facilities, long waiting lines in hospitals, financial constrains (lack of transport fares, high treatment/screening costs) and lack of health professionals to perform needed screening tests as barriers to breast cancer screening. In the recent past, gainful strides have been achieved in availing a more inclusive health insurance cover, increased adaptation of treatment guidelines, expansion and upgrading of radiotherapy equipment across the country in addition to more research opportunities [9, 21, 37]. However, even with governments efforts, the uptake of these services remains low and/or delayed over time [21, 38]. For instance, out of every seven women in Kenya, six have not been screened for breast cancer [15, 16]. The disease strikes 1 in 9 women due to late diagnosis according to the Kenya National Cancer Control strategy and as observed on burden of breast cancer and contributing factors of high mortality [23, 39–41]. Most women hardly seek professional medical attention untill symptomatic pointers are advanced [42]. It was observed in the FGDs while some urban women opted to seeking treatment in health facilities when they discovered that they had a lump in their breast, most rural women and some urban women prefered to seek care from complementary and alternative medicine providers or ignore the lump hoping it would clear off.

Against the backdrop of improvement efforts, the present findings still mirror earlier observations of low uptake and with socio-economic factors of health having been implicated in influencing individuals and communities’ health seeking behaviors [1, 43]. Further evidence that correspond to the present study points to lack of awareness, insufficient financial resources, worry about examination discomfort, fear of finding cancer due to associated myths and stigma, and inability to establish effective follow-up treatment [14, 15, 18, 20]. The barriers reported by the rural participants in the current study could be attributed to the fact that most rural areas are usually characterized by low population density and residents have poor access to health care than their urban setting counterparts as earlier observed [44]. Furthermore, according to WHO (2007) study on the social determinants of health, lack of knowledge and awareness to health is a great barrier in seeking health among women as well as stigmatization associated with breast cancer, fear, and fear of rejection by marital partners as a result of being diagnosed with the disease [45].The aspect underpins the need for family, partner involvement and both gender targeted approaches in promoting awareness which has been the effort to enhanced success of other programmes like the fight against HIV [46].

Among the urban women, decision to visit a health facility on the discovery they had breast cancer was influenced by their knowledge and information about breast cancer. Women who are knowledgeable about breast cancer and its risk factors are known to be more likely to comply with such early detection behaviors than those who are not [47]. Rural women decisions are influenced by lack of information on breast cancer with one of the leading factors to late presentation being lack of awareness about benefits of early detection of breast cancer as observed in varied setting and colloborated by the presented findings [48–51]. Consequently, there are high indications that women have misconceptions on breast cancer and its screening because they cannot access health information [12, 15]. This line of thought is reinforced by the Kenya Cancer Research and Control National Stakeholder Meeting action points that highlight: 1) Engaging community leaders and members to identify key drivers of stigma through Knowledge, Attitude, and Practice (KAP) studies, 2) Developing culturally appropriate messages to address perceptions and knowledge gaps, 3) Coordinating knowledge sharing about community education efforts and 4) Raising public awareness about cancer prevention and early detection, targeting 60% of the population by 2018. Incidences of breast cancer have been observed to be low among rural women however, death rates are higher among those diagnosed with the complication [44]. The high death rate among rural women diagnosed could be attributed to their fear and perception about the disease (it would lead to further spread, death, and loss of their position in the society as women). The presented findings on perceived severity were comparable to preceding assesesments [15, 16] despite the varied geographical and cultural settings.

## Conclusion

Creating breast cancer awareness alongside clear guidelines on accessing screening and treatment infrastructure is critical. The messaging should aim at instilling hope and eradicating the myths and misconseptions harboured about the disease. It was evident, a diagnosis of breast cancer/lump with lack of clear course of expertise support, brings unpexpected confrontation with mortality; the fear, pain, cultural barriers, emotional and financial distress are very real. Without clear referral channels to enable those with suspicious lumps or early stage disease to get prompt diagnosis and treatment, then well-meaning awareness will not necessarrily contribute to reducing morbidity and mortality.

## Additional files

Additional file 1: Interview guide for the focus group discussions and in-depth interviews (key informants) (DOCX 44 kb)

Additional file 2: Map of the study areas. (DOCX 2073 kb)

Additional file 3: Perceived barriers to early breast cancer screening uptake and treatment as mentioned by FGD participants. (DOCX 18 kb)

## Abbreviations

BSE: Breast self-examinations
CBE: Clinical breast exams
DPHN: District Public Health Nurses
FGD: Focus group discussion
HBM: Health belief model
IEC: Information, education and communications
KII: Key informant interview
NCDs: Non-communicable diseases
