# Supplementary material for: Determinants of breast cancer early detection for cues to expanded control and care: the lived experiences among women from Western Kenya
Source: BMC Womens Health. 2018 Jun 1;18:81. doi: 10.1186/s12905-018-0571-7 (PMC5984781; doi:10.1186/s12905-018-0571-7)
Supplement: Supplementary file 1 — Interview guide for the focus group discussions and in-depth interviews (key informants) (DOCX 44 kb) [file 12905_2018_571_MOESM1_ESM.docx]

# Additional file 1: Interview guide for the focus group discussions and in-depth interviews (key informants)

# ITEM 1: INTERVIEW GUIDE: FOCUS GROUP DISCUSSIONS

**Understanding of Breast Cancer and Breast Cancer Screening**

1. Discuss women’s perceptions of breast cancer and breast cancer screening (encourage women to discuss the issues by asking the following questions).

**Knowledge**

1. Which County/Constituency/Ward is this?
2. Are there any diseases that affect the breast?

Probe: If yes, what are they?

1. What does breast cancer mean to you?
2. How can breast cancer be noticed?
3. What factors may trigger breast cancer?
4. Can breast cancer be cured /prevented?

Probe: How do you prevent it /what are the treatment options available? /Where can they receive care?

1. What does breast cancer screening mean to you?

Probe: Where can you perform a breast cancer screening exercise?

Probe: How is breast cancer screening done?

1. What are the breast cancer screening exercises that you know of?
2. What are benefits of breast cancer screening?

Probe: what are the benefits to you as an individual/ what are the benefits to your family? What are the benefits to the society?

1. What are disadvantages of breast cancer screening?

Probe: What are the disadvantages to you as an Individual/to your family/to the society?

**Attitude and Health seeking behavior**

1. What do women in this community do on discovering that they have breast cancer?
2. Do women regularly undertake breast self-examination within the division as required? Give reasons for this response
3. Do the women who have suspicious lumps in their breasts go for clinical examination in MCH clinics always? Give reasons for this response?
4. How does attitude about breast cancer (such as its cause and urgency) affect breast cancer treatment?
5. How does attitude about breast cancer screening (such as its necessity) affect breast cancer screening uptake?
6. Do you know of any cultural or religious practices that affect the uptake of breast cancer screening in this community?
7. Do the following factors influence breast cancer screening uptake among women in this community

- level of education (yes/no), explain
- marital status (yes/no), explain
- distance to screening facilities, explain
- income status, explain and
- The cost of screening, explain.

1. How does fear, pain and embarrassment of women influence breast screening uptake?
2. How does the knowledge about breast cancer and breast screening influence the uptake of screening by the women? Explain.
3. What hinders you/women in this community from going for breast cancer screening?
4. For women like yourself, how often do you go for breast cancer screening?
5. How is a woman with breast cancer treated/viewed in your community
6. For women like you what would makes/motivates you to do breast cancer screening

Probe: Breast self-examination? / Clinical breast examination? /Mammogram

1. For women like yourself what makes it difficult for you to do breast cancer screening

Probe: Breast self-examination? / Clinical breast examination? /Mammogram

1. In your view, what are some suggestions that would make it easier for you to do regular breast self-examination/clinical breast examination/Mammogram?
2. What encourages or will encourage women in this community to go for breast cancer screening?
3. If you could request for some kind of services for breast cancer, what would you want?

**Sources of Information**

Discuss sources of information related to breast cancer and breast cancer screening by asking:

1. Do you have any information related to breast cancer and breast cancer screening?
2. How did you access this information on breast cancer and breast cancer screening?
3. What are barriers to breast cancer and breast cancer screening information?
4. In your view, what would be the best channel of communication of information of breast cancer and breast cancer screening?
5. Do you know of any cancer organization aimed to increase information and raise awareness for breast cancer that exists in Kenya/this community?

Probe: What are some of the breast cancer program that you know of?

**ITEM II: INTERVIEW GUIDE: IN-DEPTH INTERVIEWS (KEY INFORMANTS)**

**Background Information**

Name of Respondent…………………….............Age…………...…Constituency ………………….

Marital status ………………….. Profession ………….. Education level ………………………

**Understanding of Breast Cancer and Breast Cancer Screening**

**Knowledge**

1. Which County/Constituency/Ward is this?
2. Does the Kakamega level 5 health facility have an operational breast clinic within it? If so, state the facilities and services offered. ( if No skip to number vi)
3. Is there an operational breast health protocol used by nurses/doctors in the health facility? Explain.
4. How many women were referred from MCH clinic to the breast clinic within the last two months?
5. Does the health facility have a working mammogram /breast scan facility within it?
6. What specific aspects of breast cancer and screening if any are covered at the MCH clinic?
7. About how much time is allocated for breast health education relative to other health issues in a month within the MCH clinic? Give a reason(s) for your response.
8. Does the health facility have follow up visitation cards for women on BSE and clinical examination? Explain
9. What does breast cancer mean to you?
10. What does breast cancer screening mean to you?
11. What does breast cancer mean to the people in this community?
12. What does breast cancer screening mean to the people of this community?
13. According to the women of this community, what factors may trigger breast cancer?
14. According to the women in this community, how can breast cancer be treated?

Probe: How/what are the treatment options available

1. According to the women of this community, what are benefits of breast cancer screening?
2. According to the women of this community, what are disadvantages of breast cancer screening?

**Attitude and health seeking behavior**

1. Have you ever gone for a breast cancer screening exercise?
2. Where do women in this community go to on discovering that they have breast cancer?
3. How does attitude about breast cancer (such as its cause and urgency) and breast cancer screening (such as its necessity) affect breast cancer screening uptake?
4. Do you know of any cultural or religious practices that affect the uptake of breast cancer screening in this community?
5. Do the following factors influence breast cancer screening uptake among women in this community

- level of education (yes/no), explain
- marital status (yes/no), explain
- distance to screening facilities, explain
- income status, explain and
- The cost of screening, explain.

1. How does fear, pain and embarrassment of women influence breast screening uptake?
2. Does the knowledge about breast cancer and breast screening influence the uptake of screening by the women? Explain.
3. In your view, what are some suggestions that would make it easier and encourage women in this community to do regular breast self-examination/clinical breast examination/Mammogram?
4. What hinders the women in this community from going for clinical breast cancer screening?

**Sources of Information**

1. Have you ever accessed information on breast cancer and breast cancer screening? If yes, how? Where?
2. Do women in this community have access to information on breast cancer and breast cancer screening? If yes, how? Where?
3. Does the health facility have breast health brochures, magazines, books and videos/CDs on breast cancer and screening in the breast clinic/MCH clinic for distribution to the women? Explain
4. What are barriers to breast cancer and breast cancer screening information?
5. In your view, what would be the best channel of communication of information of cancer and breast cancer for women in this community?
6. What are some of the cancer programs aimed at increasing awareness on breast cancer that women of this community know of?
7. In your view, what are some suggestions that would make it easier for women of reproductive age in this community to undertake breast examination?

**ITEM III: DEMOGRAPHIC INFORMATION-FGDs**

1) What is your age?

___18-25

___26-35

___36-45

___46-60

2) What is your present marital status?

___Single

___Separated

___Married

___Divorced

___Widowed

3) How many children do you have? ______________________

4) What is your religious affiliation?

___Catholic

___Protestant (For example, Presbyterian, Baptist, Methodist)

___Muslim

___Non-Affiliated

___Other:______________________

(Please specify)

5) What is the highest level of education that you have?

___ None

___ Primary School

___ Secondary School

___ College/Technical School

___ University Degree

___Other:______________________

(Please specify)

6) How long have you been a resident in this community?

___ < 1 year

___ 1-2 years

___3-4 years

___4-5 years

___ > 5 years

7) What is your primary source of income?

___ Agriculture

___ Clerical

___Skilled manual labor

___Unskilled manual labor

___ Business

___ Teacher

___ Other

8) Have you ever had breast cancer screening?

___Yes

___No
