# Supplementary figures and images for: Determinants of breast cancer early detection for cues to expanded control and care: the lived experiences among women from Western Kenya
Source: BMC Womens Health. 2018 Jun 1;18:81. doi: 10.1186/s12905-018-0571-7 (PMC5984781; doi:10.1186/s12905-018-0571-7)

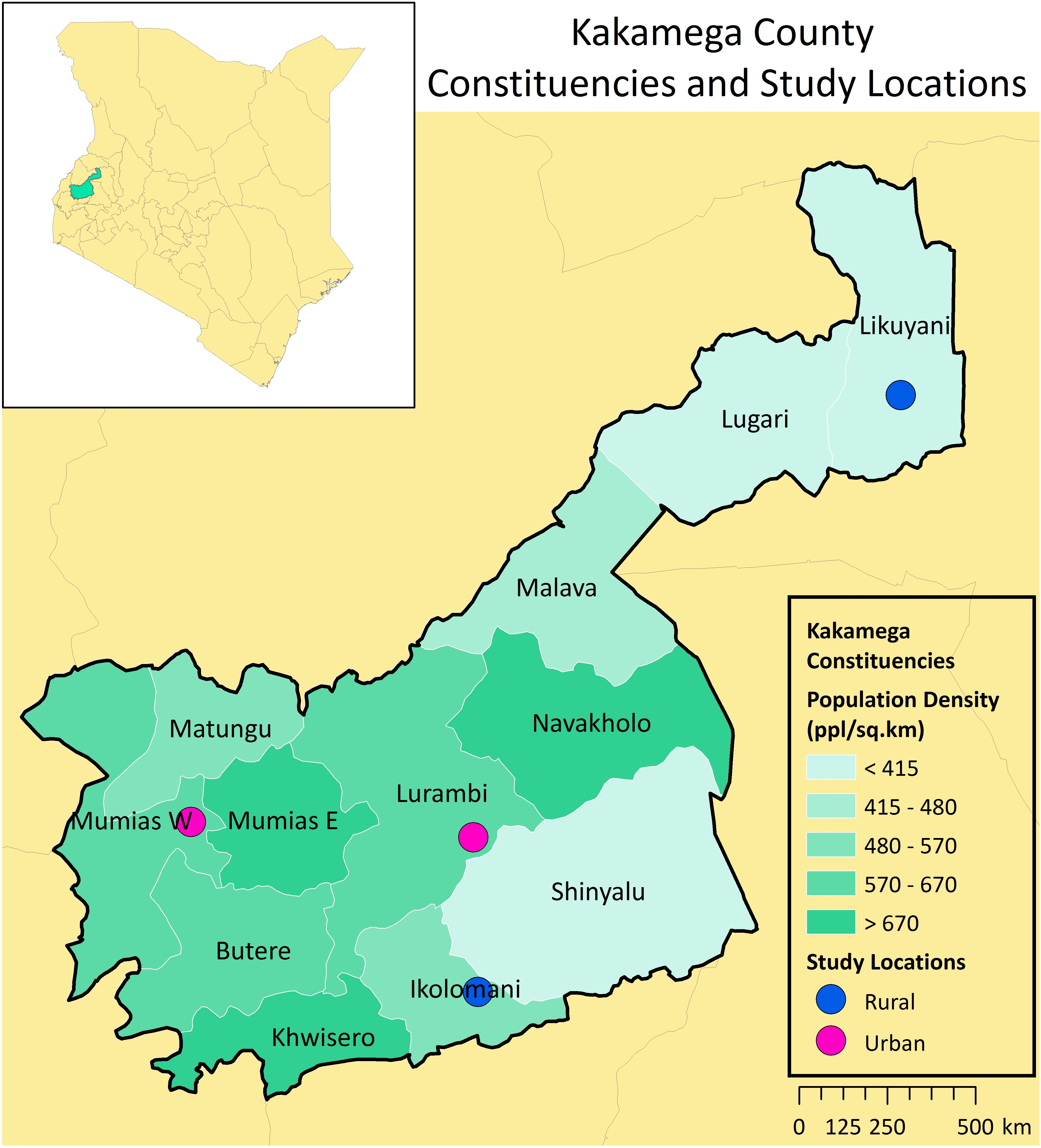


**Additional File 2: Map of the study areas**

Supplement: Supplementary file 2 — Map of the study areas. (DOCX 2073 kb) [file 12905_2018_571_MOESM2_ESM.docx]
