# Supplementary material for: Determinants of breast cancer early detection for cues to expanded control and care: the lived experiences among women from Western Kenya
Source: BMC Womens Health. 2018 Jun 1;18:81. doi: 10.1186/s12905-018-0571-7 (PMC5984781; doi:10.1186/s12905-018-0571-7)
Supplement: Supplementary file 3 — Perceived barriers to early breast cancer screening uptake and treatment as mentioned by FGD participants. (DOCX 18 kb) [file 12905_2018_571_MOESM3_ESM.docx]

**Additional file 3: Perceived barriers to early breast cancer screening uptake and treatment as mentioned by FGD participants**

| **Perceived Barriers** | **Rural** | | | | **Urban** | | | |
| --- | --- | --- | --- | --- | --- | --- | --- | --- |
|  | **Likuyani** | | **Ikolomani** | | **Lurambi** | | **Mumias West** | |
|  | Group 1 (young) | Group 2 (older) | Group 3 (young) | Group 4 (older) | Group 5 (young) | Group 6 (older) | Group 7 (young) | Group 8 (older) |
| Marital Status |  |  |  |  |  |  |  |  |
| Transport |  |  |  |  |  |  |  |  |
| Lack of Information |  |  |  |  |  |  |  |  |
| Stigma and Fear |  |  |  |  |  |  |  |  |
| Religion |  |  |  |  |  |  |  |  |
| Cultural Beliefs |  |  |  |  |  |  |  |  |

Key

| Not popular |  |
| --- | --- |
| Popular |  |
| Very popular |  |

Popularity was determined on a likert scale. If a barrier was mentioned 2 or less times in an FGD, the barrier was considered to be not popular. If a barrier was mentioned 3 to 4 times in an FGD, it was considered popular and if it was mentioned 5 or more times, it was considered as very popular.
